# Supplementary material for: Heterogeneous responses to embryonic critical period perturbations within the Drosophila larval locomotor circuit
Source: eLife. 2026 Oct 26;14:RP108656. doi: 10.7554/eLife.108656 (PMC13707376; doi:10.7554/eLife.108656)
Supplement: Supplementary file 1 [file elife-108656-mdarchecklist1.docx]

**Materials Design Analysis Reporting (MDAR)**

**Checklist for Authors**

The [MDAR framework](https://osf.io/xfpn4/) establishes a minimum set of requirements in transparent reporting mainly applicable to studies in the life sciences.

*eLife* asks authors to **provide detailed information within their article** to facilitate the interpretation and replication of their work. Authors can also upload supporting materials to comply with relevant reporting guidelines for health-related research (see [EQUATOR Network](http://www.equator-network.org/%20)), life science research (see the BioSharing Information Resource), or animal research (see the [ARRIVE Guidelines](http://www.plosbiology.org/article/info:doi/10.1371/journal.pbio.1000412) and the [STRANGE Framework](https://doi.org/10.1038/d41586-020-01751-5); for details, see *eLife*’s [Journal Policies](https://reviewer.elifesciences.org/author-guide/journal-policies)). Where applicable, authors should refer to any relevant reporting standards materials in this form.

For all that apply, please note **where in the article** the information is provided. Please note that we also collect information about data availability and ethics in the submission form.

**Materials:**

| **Newly created materials** | **Indicate where provided: section/figure legend** | **N/A** |
| --- | --- | --- |
| The manuscript includes a dedicated "materials availability statement" providing transparent disclosure about availability of newly created materials including details on how materials can be accessed and describing any restrictions on access. | Resource availability, Materials availability. No new reagents were generated. |  |
|  |  |  |
| **Antibodies** | **Indicate where provided: section/figure legend** | **N/A** |
| For commercial reagents, provide supplier name, catalogue number and [RRID](https://scicrunch.org/resources), if available. | Methods, FOS immunohistochemistry and Oxytocin immunohistochemistry; Key Resources Table (supplier, catalog number, RRID). |  |
|  |  |  |
| **DNA and RNA sequences** | **Indicate where provided: section/figure legend** | **N/A** |
| Short novel DNA or RNA including primers, probes: Sequences should be included or deposited in a public repository. | No novel sequences. Commercial RNAscope probes (*Oxt*, *Fos*, *Oxtr*; ACD Bio) and viral vectors are listed with catalog numbers in Methods and the Key Resources Table. |  |
|  |  |  |
| **Cell materials** | **Indicate where provided: section/figure legend** | **N/A** |
| Cell lines: Provide species information, strain. Provide accession number in repository OR supplier name, catalog number, clone number, OR RRID. |  | X |
| Primary cultures: Provide species, strain, sex of origin, genetic modification status. |  | X |
|  |  |  |
| **Experimental animals** | **Indicate where provided: section/figure legend** | **N/A** |
| Laboratory animals or Model organisms: Provide species, strain, sex, age, genetic modification status. Provide accession number in repository OR supplier name, catalog number, clone number, OR RRID. | Methods, Animals; Key Resources Table. Female *Mus musculus*: C57BL/6J (JAX 000664) and Oxt-IRES-Cre (JAX 024234), 8 to 20 weeks old. |  |
| Animal observed in or captured from the field: Provide species, sex, and age where possible. |  | X |
|  |  |  |
| **Plants and microbes** | **Indicate where provided: section/figure legend** | **N/A** |
| Plants: provide species and strain, ecotype and cultivar where relevant, unique accession number if available, and source (including location for collected wild specimens). |  | X |
| Microbes: provide species and strain, unique accession number if available, and source. |  | X |
|  |  |  |
| **Human research participants** | **Indicate where provided: section/figure legend) or state if these demographics were not collected** | **N/A** |
| If collected and within the bounds of privacy constraints report on age, sex, gender and ethnicity for all study participants. |  | X |

**Design:**

| **Study protocol** | **Indicate where provided: section/figure legend** | **N/A** |
| --- | --- | --- |
| If the study protocol has been pre-registered, provide DOI. For clinical trials, provide the trial registration number OR cite DOI. |  | X |
|  |  |  |
| **Laboratory protocol** | **Indicate where provided: section/figure legend** | **N/A** |
| Provide DOI OR other citation details if detailed step-by-step protocols are available. | Methods describe all procedures. Home cage recording follows Landen et al., 2024. |  |
|  |  |  |
| **Experimental study design (statistics details) *** | | |
| **For in vivo studies: State whether and how the following have been done** | **Indicate where provided: section/figure legend. If it could have been done, but was not, write “not done”** | **N/A** |
| Sample size determination | Prospective power analyses (G*Power and R; alpha = 0.05, power = 0.80) were performed for fiber photometry and optogenetic experiments as part of the IACUC approved protocol. Photometry was powered on an effect size of 1.06 for the change in oxytocin neuron firing during social interaction (Tang et al., 2020). Optogenetics was powered on an expected core body temperature difference of 1.25 °C between ChR2 and light only stimulation (SD 0.8; Fukushima et al., 2022). Both analyses planned 8 mice per group. Formal power analyses were not performed for FOS immunohistochemistry, RNAscope, or lactation experiments. Sample sizes were similar to those of previous studies (Tang et al., 2020; Yukinaga et al., 2022; Carcea et al., 2021). Analyzed sample sizes are reported in each figure legend and Supplementary File 1. |  |
| Randomisation | Photometry condition order (floor temperature and social context) was pseudorandomized (Figure 2C). FOS groups were defined by spontaneous behavior. Allocation of mice to ChR2+ or light only control groups was not randomized (not done). |  |
| Blinding | Not done. Experimenters were not blinded to condition during data collection or manual behavioral annotation. To limit observer bias, FOS counts, calcium peak detection, and SGBS temperature extraction were automated with fixed parameters (Methods). |  |
| Inclusion/exclusion criteria | Methods: mice without GCaMP signal were excluded; fiber placement was confirmed histologically with no exclusions; peaks were defined as at least 6 SD above mean (7 SD for lactation); bouts under 2 min were excluded from Tb regression (Figure 3–figure supplement 1). |  |
|  |  |  |
| **Sample definition and in-laboratory replication** | **Indicate where provided: section/figure legend** | **N/A** |
| State number of times the experiment was replicated in the laboratory. | Animal and recording numbers are in each figure legend and Supplementary File 1. |  |
| Define whether data describe technical or biological replicates. | Biological replicates are mice. Recordings, bouts, peaks, stimulations, and ROIs are repeated measures within mice, modeled with mouse ID as a random effect (Methods, Statistics). |  |
|  |  |  |
| **Ethics** | **Indicate where provided: section/submission form** | **N/A** |
| Studies involving human participants: State details of authority granting ethics approval (IRB or equivalent committee(s), provide reference number for approval. |  | X |
| Studies involving experimental animals: State details of authority granting ethics approval (IRB or equivalent committee(s), provide reference number for approval. | Methods, Animals. University of Wyoming IACUC, protocol 2025-0206 (continuation of protocol 20220217AN00537-01). |  |
| Studies involving specimen and field samples: State if relevant permits obtained, provide details of authority approving study; if none were required, explain why. |  | X |
|  |  |  |
| **Dual Use Research of Concern (DURC)** | **Indicate where provided: section/submission form** | **N/A** |
| If study is subject to dual use research of concern regulations, state the authority granting approval and reference number for the regulatory approval. |  | X |

**Analysis:**

| **Attrition** | **Indicate where provided: section/figure legend** | **N/A** |
| --- | --- | --- |
| Describe whether exclusion criteria were pre-established. Report if sample or data points were omitted from analysis. If yes, report if this was due to attrition or intentional exclusion and provide justification. | Exclusion criteria are listed in Methods (see Inclusion/exclusion criteria). |  |
|  |  |  |
| **Statistics** | **Indicate where provided: section/figure legend** | **N/A** |
| Describe statistical tests used and justify choice of tests. | Methods, Statistics; figure legends; Supplementary File 1. Linear mixed models with mouse ID as a random effect account for repeated measures. Also used: linear regression with Tukey and Holm correction, logistic and Poisson regression, likelihood ratio test, and cross correlation. |  |
|  |  |  |
| **Data availability** | **Indicate where provided: section/submission form** | **N/A** |
| For newly created and reused datasets, the manuscript includes a data availability statement that provides details for access (or notes restrictions on access). | Resource availability, Data and code availability. |  |
| When newly created datasets are publicly available, provide accession number in repository OR DOI and licensing details where available. | OSF: https://osf.io/x9kcj |  |
| If reused data is publicly available provide accession number in repository OR DOI, OR URL, OR citation. |  | X |
|  |  |  |
| **Code availability** | **Indicate where provided: section/figure legend** | **N/A** |
| For any computer code/software/mathematical algorithms essential for replicating the main findings of the study, whether newly generated or re-used, the manuscript includes a data availability statement that provides details for access or notes restrictions. | Resource availability, Data and code availability; Methods, Computer vision analysis. |  |
| Where newly generated code is publicly available, provide accession number in repository, OR DOI OR URL and licensing details where available. State any restrictions on code availability or accessibility. | OSF: https://osf.io/x9kcj; GitHub: https://github.com/j-landen/SGBS and https://github.com/j-landen/seq_process. |  |
| If reused code is publicly available provide accession number in repository OR DOI OR URL, OR citation. | ThermImageJ (Tattersall, 2019; doi:10.5281/zenodo.2652896). Software and versions are in the Key Resources Table. |  |

**Reporting:**

The MDAR framework recommends adoption of discipline-specific guidelines, established and endorsed through community initiatives.

| **Adherence to community standards** | **Indicate where provided: section/figure legend** | **N/A** |
| --- | --- | --- |
| State if relevant guidelines (e.g., ICMJE, MIBBI, ARRIVE, STRANGE) have been followed, and whether a checklist (e.g., CONSORT, PRISMA, ARRIVE) is provided with the manuscript. | Animal procedures followed NIH guidelines (Methods, Animals). No ARRIVE or other reporting checklist is provided. |  |

* We provide the following guidance regarding transparent reporting and statistics; we also refer authors to [Ten common statistical mistakes to watch out for when writing or reviewing a manuscript](https://doi.org/10.7554/eLife.48175).

**Sample-size estimation**

- You should state whether an appropriate sample size was computed when the study was being designed
- You should state the statistical method of sample size computation and any required assumptions
- If no explicit power analysis was used, you should describe how you decided what sample (replicate) size (number) to use

**Replicates**

- You should report how often each experiment was performed
- You should include a definition of biological versus technical replication
- The data obtained should be provided and sufficient information should be provided to indicate the number of independent biological and/or technical replicates
- If you encountered any outliers, you should describe how these were handled
- Criteria for exclusion/inclusion of data should be clearly stated
- High-throughput sequence data should be uploaded before submission, with a private link for reviewers provided (these are available from both GEO and ArrayExpress)

**Statistical reporting**

- Statistical analysis methods should be described and justified
- Raw data should be presented in figures whenever informative to do so (typically when N per group is less than 10)
- For each experiment, you should identify the statistical tests used, exact values of N, definitions of center, methods of multiple test correction, and dispersion and precision measures (e.g., mean, median, SD, SEM, confidence intervals; and, for the major substantive results, a measure of effect size (e.g., Pearson's r, Cohen's d)
- Report exact p-values wherever possible alongside the summary statistics and 95% confidence intervals. These should be reported for all key questions and not only when the p-value is less than 0.05.

**Group allocation**

- Indicate how samples were allocated into experimental groups (in the case of clinical studies, please specify allocation to treatment method); if randomization was used, please also state if restricted randomization was applied
- Indicate if masking was used during group allocation, data collection and/or data analysis
