# Supplementary material for: Heterogeneous responses to embryonic critical period perturbations within the Drosophila larval locomotor circuit
Source: eLife. 2026 Oct 26;14:RP108656. doi: 10.7554/eLife.108656 (PMC13707376; doi:10.7554/eLife.108656)
Supplement: Peer review history

# Editor report: eLife Assessment

**DOI:** 10.7554/eLife.108656.3.sa0

**Authors:** Dion K Dickman

**Affiliations:** University of Southern California United States

**Keywords:** Convincing, Important

This is an **important** study of critical period plasticity, focused on temperature manipulations, and how different parts of the *Drosophila* larval motor circuit adapt or maladapt. The work **convincingly** demonstrates that components of the motor network respond in distinct ways to heat shocks, and the combination of functional, structural, and electrophysiological approaches makes the study of significant interest. The work points to central interneurons as primary drivers of maladaptive changes, while motoneurons and neuromuscular junctions show compensatory or homeostatic adjustments. The authors have thoughtfully and substantially addressed the concerns raised in the original reviews through new temporal-window experiments, additional receptor and behavioral analyses, clearer discussion of the study's limitations, and an integrative model.

# Referee report: Reviewer #1 (Public review)

**DOI:** 10.7554/eLife.108656.3.sa1

Summary:

The authors examine the impact of heat stress during an embryonic CP in *Drosophila*, focusing on the larval locomotor network. They show that elevated temperature increases neuronal activity and, when applied during the CP, results in long-term instability of the network which manifests in prolonged seizure recovery times. At the neuromuscular junction, substantial structural changes occur, including terminal overgrowth and altered receptor composition, yet synaptic transmission remains preserved due to homeostatic regulation. Motoneurons display reduced excitability but receive increased synaptic input from premotor interneurons. These findings suggest that maladaptive instability originates within the central circuitry rather than at the neuromuscular junction, where changes seem to be homeostatically compensated. The study concludes that different network components exhibit distinct and hierarchical responses to CP perturbations, with premotor interneurons setting the tone for downstream adjustments in motoneurons.

Strengths:

The work takes advantage of the unique accessibility of the *Drosophila* system. A major strength of the study is the integration of structural, physiological, and behavioral analyses, which allows the authors to draw a comprehensive picture of how CP perturbations shape the locomotor network. The choice of an ecologically relevant stimulus (heat stress) is particularly convincing, as it links experimental manipulations more closely to natural environmental conditions. The experiments are carefully designed, and the results are robust and consistent with previous findings in the field, while also extending them in new directions. Importantly the work clarifies how temperature perturbations within distinct developmental time windows affect different properties of motor circuit formation.

Weaknesses:

A small limitation of the study is that it remains difficult to integrate maladaptive (seizure recovery) and adaptive/homeostatic phenotypes within a single mechanistic framework, leaving some space for interpretation.

Comments on revised version.

I think the authors did a great job at revising the manuscript and they addressed all my comments.

# Referee report: Reviewer #2 (Public review)

**DOI:** 10.7554/eLife.108656.3.sa2

Summary:

This manuscript presents a thoughtful and well-executed study of critical period plasticity in the *Drosophila* larval motor circuit. The authors examined how transient heat, 32C, during embryonic stage, altered network properties, showing that premotor interneurons A27h increase excitatory drive onto motoneurons, which respond with a reduction in excitability. At the NMJ, synaptic terminals expand and GluRIIA distribution shifts, yet synaptic transmission remains largely unaffected. Despite these local compensations, the treated larvae display slower crawling and prolonged recovery from seizures, indicating that the network is functionally compromised.

Strengths:

(1) One of the major strengths of this study is the elegant dissection of a defined circuit, tracking changes from premotor interneurons through motoneurons to the NMJ. The multimodal approach provides a comprehensive view of how connected elements respond to CP perturbations.

(2) An interesting finding is that NMJ morphology changes dramatically without corresponding deficits in synaptic transmission, challenging the common assumption that larger boutons necessarily indicate stronger synapses.

(3) Another intriguing result is that even with two layers of homeostatic compensation, locomotor behavior is still impaired, highlighting the limits of compensation and underscoring the critical role of CP timing.

(4) Beyond these scientific insights, the study benefits from a well-defined, tractable system and simple experimental manipulations, which together make the results highly interpretable and reproducible.

Comments on revised version.

The authors have carefully considered my comments and recommendations and have made substantial efforts to improve the clarity and validity of the study. Although additional electrophysiology experiments using shorter heat stress windows were not feasible, the authors performed additional analyses of postsynaptic GluRs and provided a clearer discussion of the study's limitations. Overall, this is a strong and well-written paper that establishes a valuable foundational framework for addressing interesting and important questions about adaptive responses in developing neural circuits.

# Author comment: Author response

**DOI:** 10.7554/eLife.108656.3.sa3

**Authors:** Niklas Krick, Jacob J Davies, Bramwell Coulson, Daniel Sobrido-Cameán, Michael Miller, Matthew CW Oswald, Aref A Zarin, Richard A Baines, Matthias Landgraf

**Affiliations:** University of Cambridge Cambridge United Kingdom; University of Manchester Manchester United Kingdom; University of Manchester Manchester United Kingdom; University of Cambridge Cambridge United Kingdom; University of Cambridge Cambridge United States; University of Cambridge Cambridge United Kingdom; Texas A &M University College Sttaion United States; University of Manchester Manchester United Kingdom; University of Cambridge Cambridge United Kingdom

The following is the authors’ response to the original reviews.

**eLife Assessment:**

This is an important study of critical period plasticity, focused on temperature manipulations, and how different parts of the *Drosophila* larval motor circuit adapt or maladapt. The work convincingly demonstrates that components of the motor network respond in distinct ways to the heat shock, and the combination of functional, structural, and electrophysiological approaches makes the study of significant interest. The work points to central interneurons as primary drivers of maladaptive changes, while motoneurons and neuromuscular junctions show compensatory or homeostatic adjustments. The study is methodologically rigorous, contributing important insights into critical period biology using a tractable invertebrate model.

We thank the reviewers for their thoughtful critique and suggestions. We agree with these and, where possible, we have attempted to address these, improving this study. As outlined below, we have revised the manuscript substantively and included additional data and figures.

**Public Reviews:**

**Reviewer #1 (Public review):**

Summary:

The authors examine the impact of heat stress during an embryonic CP in *Drosophila*, focusing on the larval locomotor network. They show that elevated temperature increases neuronal activity and, when applied during the CP, results in long-term instability of the network, which manifests in prolonged seizure recovery times. At the neuromuscular junction, substantial structural changes occur, including terminal overgrowth and altered receptor composition, yet synaptic transmission remains preserved due to homeostatic regulation. Motoneurons display reduced excitability but receive increased synaptic input from premotor interneurons. These findings suggest that maladaptive instability originates within the central circuitry rather than at the neuromuscular junction, where changes seem to be homeostatically compensated. The study concludes that different network components exhibit distinct and hierarchical responses to CP perturbations, with premotor interneurons setting the tone for downstream adjustments in motoneurons.

Strengths:

The work takes advantage of the unique accessibility of the *Drosophila* system. A major strength of the study is the integration of structural, physiological, and behavioral analyses, which allows the authors to draw a comprehensive picture of how CP perturbations shape the locomotor network. The choice of an ecologically relevant stimulus (heat stress) is particularly convincing, as it links experimental manipulations more closely to natural environmental conditions. The experiments are carefully designed, and the results are robust and consistent with previous findings in the field, while also extending them in new directions.

Weaknesses:

The study leaves some uncertainty regarding the experimental design and interpretation. The change from short to prolonged heat shock manipulations raises the possibility that the effects observed may not be confined to the critical period alone - this could be experimentally addressed or simply rephrased in the text.

We agree that clarity about the experimental paradigm is important and have addressed this as suggested, within text, figures and figure legends: the duration of embryo exposure to 32°C heat stress is now unambiguously stated and each figure has a graphical illustrations of the heat stress paradigm. For example, experiments represented in Figures 1, 3 (new data) and 8 (new data) used short, defined periods of a few hours of heat stress, aimed to identify specific windows of development that are sensitive to 32°C heat stress. These also show that behavioural changes result from heat stress experienced during the specific 2-hour window that defines the critical period of the developing central locomotor circuitry, namely from 17-19 hours after egg laying - previously identified by Giachello & Baines (2015). Longer exposure to 32°C heat stress during embryogenesis result in the same phenotypes when this 2-hour window is included, causing the same level of reduced larval crawling speed and lowered network stability, which manifests in increased seizure recovery times. This is as one might expect from a critical period of nervous system development.

Where the neuromuscular junction is concerned, where we identified embryonic heat stress causing phenotypes that are evident at late larval stages, a more complex model has emerged. Following suggestions from both reviewers to explore shorter heat stress exposures during embryogenesis, additional experiments (see Figure 3) we identified what might be a critical period for the body wall muscles. This is an earlier window of development, within 13-16 hours after egg laying, which is sensitive to heat stress in terms of the levels of the GluRIIA glutamate receptor subunit that will be expressed in the late larva. This developmental period is characterised by muscles acquiring their electrical properties (Broadie & Bate, 1993), i.e. comparable to the central locomotor network transitioning through its critical period at the time that it becomes active. The neuromuscular junction is composed of both presynaptic motoneurons and postsynaptic muscles, and therefore this composite structure is subject to multiple, sequential critical periods. The characterisation of changes to neuromuscular junction synaptic physiology was carried out using heat stress throughout most of embryogenesis (Figure 4). We think this appropriate from the perspectives of having included all relevant critical periods (muscle and CNS) to explore how this composite structure responds to environmental heat stress; also based on our observations that for each critical period phenotypes are defined by the experience during the critical period and not exacerbated by prolonged heat stress either side.

In addition, the maladaptive (seizure recovery) and adaptive/homeostatic phenotypes are not always clearly distinguished or highlighted, which makes it harder to appreciate how the different levels of the network plasticity fit together into a single mechanistic framework.

Following the suggestion, we have tried to clarify the mechanistic framework in a new figure that aims to summarise the model in Figure 9.

The question of whether phenotypes that result from an embryonic heat stress manipulation are adaptive or maladaptive is difficult to resolve. This is partly due to the nature of critical periods, since perturbations during these developmental windows can cause significant, long-lasting maladaptations that are challenging to reconcile from a perspective of adaptive plasticity. Secondly, in light of the nature of this animal, which has evolved a particularly rapid development and large brood sizes, any deviation from the evolved optimum developmental temperature of 25 °C could constitute a reduction in fitness. We interpret the phenotypes we see along those lines: network instability that results from critical-period perturbations is a manifestation of a sub-optimally tuned network, as is a reduction in larval crawling speed.

**Reviewer #2 (Public review):**

Summary:

This manuscript presents a thoughtful and well-executed study of critical period plasticity in the *Drosophila* larval motor circuit. The authors examined how transient heat, 32 {degree sign}C, during the embryonic stage, altered network properties, showing that premotor interneurons A27h increase excitatory drive onto motoneurons, which respond with a reduction in excitability. At the NMJ, synaptic terminals expand and GluRIIA distribution shifts, yet synaptic transmission remains largely unaffected. Despite these local compensations, the treated larvae display slower crawling and prolonged recovery from seizures, indicating that the network is functionally compromised.

Strengths:

(1) One of the major strengths of this study is the elegant dissection of a defined circuit, tracking changes from premotor interneurons through motoneurons to the NMJ. The multimodal approach provides a comprehensive view of how connected elements respond to CP perturbations.

(2) An interesting finding is that NMJ morphology changes dramatically without corresponding deficits in synaptic transmission, challenging the common assumption that larger boutons necessarily indicate stronger synapses.

(3) Another intriguing result is that even with two layers of homeostatic compensation, locomotor behavior is still impaired, highlighting the limits of compensation and underscoring the critical role of CP timing.

(4) Beyond these scientific insights, the study benefits from a well-defined, tractable system and simple experimental manipulations, which together make the results highly interpretable and reproducible.

Weaknesses:

There are a few areas where the manuscript could be strengthened.

(1) Although A27h premotor neurons are well characterized, the claim that they are the causal driver of downstream changes would be strengthened by additional experiments or a clearer discussion of the temporal hierarchy.

We have tried to clarify the model of the temporal hierarchy (new Figure 9). This is a model and as such will hopefully help us collectively to think about this system and underlying processes, while also inviting this perspective to be challenged. The model we propose is compatible with our observations, namely that the premotor circuitry might change in response to a critical period heat stress (e.g. increasing their synaptic drive onto motoneurons), followed by homeostatic adjustment by the postsynaptic motoneurons (e.g. by reduction of their excitability), thus serving to maintain overall normal motoneuron firing patterns (see Figure 6).

However, synaptic communication is commonly regulated in both antero- and retrograde directions. Therefore, while compatible with the observations we have made, bi-directional information flow could also be instructive during the CNS critical period.

(2) While 32 {degree sign}C heat stress is presented as ecologically relevant, it produces maladaptive behavioral outcomes, raising questions about the ecological and mechanistic interpretation of the model. In particular, most experiments, with the exception of Figure 1, used prolonged (24h) heat treatments, which could introduce developmental effects beyond the CP itself. Comparing shorter and longer heat exposures would help clarify the specificity of the CP response.

We agree. For a detailed response on this point, please response to Reviewer #1 above.

(3) While there are schematics for experimental procedures, a circuit diagram tracing information flow and indicating where structural and functional changes occur would help readers better understand the findings.

We have created Figure 9 as a working model.

(4) Finally, the main paradox of the study, that robust homeostatic compensations occur yet behavior remains impaired, could be explored in more depth in the Discussion.

We have tried to address this in the discussion.

**Reviewer #3 (Public review):**

Summary:

During development, neural circuits undergo brief windows of heightened neuronal plasticity (e.g., critical periods) that are thought to set the lifelong functional properties of underlying circuits. These authors, in addition to others within the *Drosophila* community, previously characterized a critical period in late fly embryonic development, during which alterations to neuronal activity impact late-stage larval crawling behavior. In the current study, the authors use an ethologically-relevant activation paradigm (increased temperature) to boost motor activity during embryogenesis, followed by a series of electrophysiology and imaging-based experiments to explore how 3 distinct levels of the circuit remodel in response to increases in embryonic motor activity. Specifically, they find that each level of the circuit responds differently, with increased excitatory drive from excitatory pre-motor neurons, reduced excitability in motor neurons, and no physiological changes at the NMJ despite dramatic morphological differences. Together, these data suggest that early life experience in the motor neuron drives compensatory changes at each level of the circuit to stabilize overall network output.

Strengths:

The study was well-written, and the data presented were clear and an important contribution to the field.

Weaknesses:

The sample sizes and what they referred to throughout the distinct studies were unclear. In the legends, the authors should clearly state for each experiment N=X, and if N refers to an NMJ, for example, instead of an individual animal, they should state N=X NMJs per N=X animals. This will help readers better understand the statistical impact of the study.

This is a good point. For the majority, each data point is derived from a unique specimen, unless explicitly stated otherwise, for NMJ size on muscle DA1 (Figure 3) and for larval crawling data, where each larva was measured up to three time, once per unique 5-minute crawling interval.

**Recommendations for the authors:**

**Reviewing Editor Comments:**

In addition to revising the text and making interpretive changes as suggested by the reviewers, we invite you to consider the following:

(1) Either rephrase the conclusions on the role of the 2h CP and discuss the effects of temperature during embryonic development. Alternatively, to validate the idea of a longer CP window, directly compare the results of a few key experiments using the 2h and 24h heat treatment.

We have addressed this within the text, as suggested. In the text and figure legends, we have made clear distinctions between exposure to 32°C heat stress during most of embryogenesis vs a specific developmental window of a few hours. In figures, we have provided diagrams that graphically illustrate the period of heat stress exposure.

Our ability to experimentally test differences between precise vs broader heat stress periods during embryonic development have been constrained due to the departure of scientists, who were able to carry out electrophysiological recordings (as also explained below). As the next best alternative, we focus on imaging and behavioural analyses. These demonstrated that the developing body wall muscles are sensitive to heat stress during an earlier phase, from 13-16 hours after egg laying, when the body wall muscles become electrically active. It precedes the critical period of the central locomotor circuitry (17-19 hours after egg laying), when neurons in the CNS become electrically and synaptically active (16 hours after egg laying). We think this an exciting additional insight, demonstrating sequential critical periods as different parts of the locomotor network become active: first the body wall muscles, followed by the central circuitry.

(2) Clarifying the homeostatic responses and shedding light on how they engage with the maladaptive changes described would benefit the study. Furthermore, adding more information about the anatomical and structural changes and how they relate to the intrinsic and synaptic changes would also benefit the study.

We have tried to address this within the text and with a summary diagram (Figure 8), as suggested.

**Reviewer #1 (Recommendations for the authors):**

(1) It remains unclear whether the authors want to conclude that reduced network stability is not due to changes at the motoneuron level, but rather at the premotor level. Although this idea is mentioned in the results and discussion, it does not appear in the abstract or introduction, which leaves the different findings disconnected. Clarifying and highlighting this conclusion throughout the manuscript would strengthen the narrative.

We have added additional experiments and changed the manuscript to address this point. These showed that there are distinct phases of embryonic development during which heat stress causes changes to NMJ structure vs to larval behaviour (seizure recovery times/network stability and crawling speed) - additional data in suppl. Fig. 2 and Fig. 7. In the *Drosophila* embryo, the body wall muscles develop and acquire their electrical properties before central neurons do, and these phases correlate with sensitivity to heat stress.

(2) In the results section related to Figure 1, the logical link between the CP protocol and the functional assessment of the locomotor network at different temperatures is not sufficiently explained. It is not clear what this assessment is meant to test or demonstrate. A more explicit statement of the rationale and correction of what seems to be a typographical error in the final sentence of the paragraph would help to clarify the authors' intent.

We have tried to rectify this by changes in the manuscript and to Figure 1, to make the sequence of panels more intuitive.

(3) In the second results section, the experimental strategy shifts from using a short 2-hour heat shock to a 24-hour manipulation. The reasoning - that short manipulations in different windows yield no phenotype - is understandable, but a 24-hour perturbation may have broader consequences beyond the CP, simply by virtue of its longer duration. Moreover, 24h is roughly the duration of embryonic development at 25C. When at 32C, embryos should develop faster; therefore, is the 24h heat shock extending to L1?

Yes, the 24 hour heat stress extends into the first few hours of the L1 larval stage.

In order to validate the use of a longer window, the author should show how it affects the developmental time. Moreover, one should test that a few important observations remain the same with 2h and 24h heat perturbation. Alternatively, one cannot conclude that the phenotype is due to the rather narrow previously defined CP rather than to other effects associated with the overall embryonic developmental time and coordination. This would not make the results less interesting, but it would be important to assess whether the effects can be solely attributed to the 2h CP.

We have compared the impact of heat stress experience during the majority of embryogenesis, including the CP that had been defined for the central locomotor network (17-19 hours after egg laying) with shorter heat stress manipulations during consecutive phases of embryogenesis until larval hatching. As outlined above in response to point (1) by the Reviewing Editor, reduced stability of the central network and associated reduction in larval crawling occurs when heat stress is experienced during the CP of the central locomotor network (17-19 hours after egg laying). Prolonged heat stress experience for 24 hours leads to indistinguishable outcomes, as long as this 2-hour CP window is included (see Fig. 1 and Fig. 8).

However, this suggestion by Reviewer #1 led us to identify a second CP for the body wall muscles (see Fig. 3). NMJ overgrowth and changes to the postsynaptic glutamate receptor composition result from earlier heat stress experiences, and those are comparable to the effects caused by 24-hour heat stress exposure when this earlier muscle CP is included.

Therefore, NMJ development is affected by consecutive CPs, an earlier one linked to body wall muscle development, followed by a later one that impacts the presynaptic motoneurons and their upstream circuitry. Nevertheless, the larval NMJ and behavioural phenotypes that we have identified appear to result from sensitivity to heat stress during these respective CP windows, with no clear evidence of cumulative effects on these phenotypes resulting from longer heat stress exposure during embryogenesis.

(4) In session 3, the authors note that GluRIIA reductions were most pronounced in proximal regions of the NMJ. However, this is not explicitly quantified in the figures or methods. Including such quantification, or clarifying where it can be found, would make this observation more convincing.

We have analysed anti-GluRIIA signal intensities in proximal vs distal boutons, comparing different ROI selection processes (e.g. thresholding to a full NMJ/anti-HRP mask and to an anti-GluRIIB mask, which is more selective to postsynaptic sites). Analysis of multiple data sets did not show statistical significance, but instead confirmed that comparable reductions in anti-GluRIIA signal manifest in both proximal and distal boutons, following an embryonic 32C heat stress, relative to controls. We have therefore removed relevant speculative statements.

(5) In session 4, the authors conclude that motoneurons undergo a decrease in excitability to adjust to greater premotor drive. Is there anatomical evidence for this, such as an increase in input synapses?

We previously quantified change in excitatory presynaptic synaptic contact number onto aCC motoneuron dendrites in third instar larvae following an embryonic pharmacological activity manipulation: overexcitation of the developing network following introduction of PTX via feeding to gravid females*. No significant structural changes were seen. Although this is a different manipulation of the developing network, all our data to date suggest that heat stress manipulations during the embryonic critical period signal via the same pathways, at least in part due to temperature increases leading to activity increases. Because such a quantification is technically challenging and extremely time-consuming due to the low level of marking individual motoneurons, we did not think it informative or in scope for this project.

*See Figure 5 in this publication: Hunter I, Coulson B, Pettini T, Davies JJ, Parkin J, Landgraf M, Baines RA. Balance of activity during a critical period tunes a developing network. Elife. 2024 Jan 9;12:RP91599. doi: 10.7554/eLife.91599. PMID: 38193543; PMCID: PMC10945558.

The interpretation of the optogenetic experiments would also benefit from clarification. If motoneurons are less excitable yet receive more drive, one might expect no net change, rather than the differences observed. Alternatively, could the excitability of the premotor neuron itself have changed, either intrinsically or in relation to Chronos expression? Measuring premotor activity directly during optogenetic activation could help to resolve this ambiguity.

These are good suggestions. Yes, we think that motoneuron excitability has changed as a result of heat stress - see paper submitted in parallel and published since: Sobrido-Cameán et al., 2025, PLoS Biology. Unfortunately, the team members, who could have carried out this type of analysis had moved on by submission of the manuscript. Therefore, we were no able to experimentally pursue these questions further.

(6) In session 6, the authors report slower propagation of premotor activity waves after CP heat stress, but the logic of the experiment is not sufficiently explained. How does this finding relate to the enhanced premotor drive described earlier? Only timing is quantified; information about amplitude and wave dynamics would strengthen the interpretation. These results could also be discussed in relation to Figure 1B, where acute heat stress increased motoneuron activity. One interesting possibility could be that CP manipulations might adaptively prepare the larva to function at different temperatures. Experiments testing wave propagation at 32 {degree sign}C (Figure 6) or, conversely, motoneuron activity after CP manipulations (Figure 1B paradigm) would provide valuable evidence for such an adaptive role.

As per above, unfortunately, the team member who could have carried out this type of analysis had moved on by submission of the manuscript. However, we have tried to address the question of whether there is an adaptive element to the adjustments that result from embryonic heat stress experience. Specifically, we carried out behavioural tests on how larvae respond with changes in crawling speed to acute changes in ambient temperature (new Fig. 7).

We interpret our findings as follows: that heat stress during embryonic development leads to sub-optimal outcomes with regard to network stability as well as default and maximum crawling speed. The precise causes for this will be difficult to unpick. Behaviourally, when challenging larvae with an acute change in ambient temperature, we saw that slow crawling larvae do respond comparatively normally to a relative increase in ambient temperature by speeding up (in effect an escape response). This demonstrates that embryonic heat stress causes a change in the default crawling speed, while principally maintaining behavioural responses to changes in ambient temperature. It appears that animals that had experienced heat stress during embryonic development, by adjusting their default speed downward, maintain a dynamic response range into a higher temperature range than controls (35C vs 29C, respectively). Potentially, this could be an adaptive outcome to living at higher temperatures, though such an interpretation would require a body of work.

Nevertheless, every aspect we have assayed suggests that heat stress experience during the CP leads to sub-optimal outcomes: of network instability, slower default and slower maximal crawling speeds.

Minor points:

(1) Abstract: "has suboptimal outcomes;" should be corrected to "has suboptimal outcomes,".

Corrected

(2) Abstract: "we find that transient embryonic..." would improve readability with a capitalized "We".

We are unsure about the sentence this refers to. If this sentence, then we suggest that this could remain as was.

"Within the central nervous system, we find transient embryonic CP perturbation leads to increased synaptic drive from premotor interneurons to motoneurons..."

The intention here is to differentiate between changes within the CNS vs at the NMJ.

(3) Abstract: The sentence "Present the larva ... as an experimental model system..." overstates novelty, as the system has already been established in prior work. Instead, this study could highlight temperature manipulation as an ecologically relevant way to probe CPs.

We have adopted this suggestion.

**Reviewer #2 (Recommendations for the authors):**

I would recommend:

(1) Perform additional experimental support and dissuasions for the causal role of the premotor neuron and the network disability.

Unfortunately, it has not been possible to carry out additional e-phys experimental work due to key people having moved on and now unable to carry out such experiments, and no replacements in sight to do so. Instead, we have focused on other work that we could do, namely to test the effect of different heat stress windows during embryonic development on GluRIIA vs GluRIIB expression at postsynaptic sites. This shows that indeed the effect seen following a 24-hour heat stress is replicated by a much shorter window of heat stress. For NMJs GluRII composition the critical period is different from the critical period of the CNS. This replicates the different developmental timings of maturation: the body wall muscles express ion channels and attain their electrical properties several hours before central neurons. We have provided these additional data as a new supplementary figure to Fig. 2. We have changed the main text to note the caveat of longer heat stress manipulations potentially leading to additional or more exacerbated phenotypes.

(2) I would suggest that the authors expand the discussion on why two layers of homeostatic adjustment fail to preserve behavior. Is this simply a limit of plasticity?

This is a difficult aspect to address well, beyond the purely speculative and potentially confusing. We would like to suggest that to do so requires a basic understanding on what pressures neurons/networks respond to (heat-caused over-activation and/or metabolic); and from that perspective to gauge how they adjust to those pressures, i.e. what the adjustments are trying to "achieve". This in turn should inform on whether such adjustments are homeostatic or anti-homeostatic in nature, and whether there are limits beyond which we consider a system "breaking".

(3) 32C heat was described as "ecologically relevant". However, it produces maladaptive outcomes. The author should consider reframing it as a stressor that reveals CP sensitivity, rather than an adaptive signal.

This is a good suggestion, and we have implemented changes accordingly.

(4) It is important to distinguish the effects of transient (2h) vs prolonged heat exposure to confirm the manipulation targeted CP specifically.

We have tried to make these distinctions clearer within text and figure legends. As per response to (1) above, we generated and analysed additional data to show that changes in GluRIIA are induced during a defined shorter developmental time window, not exacerbated by prolonged heat stress exposure during embryogenesis.

(5) I would definitely recommend adding a diagram tracking information flow and showing where structural and functional changes occur.

This is a helpful suggestion, which we have tried to implement with a new figure (Fig. 8).

Overall, this is a strong and well-written paper that produced some unexpected results, and added a solid model circuit to study CP plasticity at the circuit level.

**Reviewer #3 (Recommendations for the authors):**

I identified one typo: "activity manipulations during the embryonic CP are artificial, We asked to what extent" The "W" of "We" should be lower case.

Now corrected.
